# Preliminary investigation and application of a novel deformable PRESAGE^®^ dosimeter

**DOI:** 10.1088/1742-6596/444/1/012080

**Published:** 2013

**Authors:** T Juang, J Newton, S Das, J Adamovics, M Oldham

**Affiliations:** 1 Duke University Medical Center, Durham, NC, USA; 2 Rider University, Lawrenceville, NJ, USA

## Abstract

Deformable 3D dosimeters have potential applications in validating deformable dose mapping algorithms. This study evaluates a novel deformable PRESAGE^®^ dosimeter and its application toward validating the deformable algorithm employed by VelocityAI. The deformable PRESAGE^®^ dosimeter exhibited a linear dose response with a sensitivity of 0.0032 ΔOD/(Gy/cm). Comparison of an experimental dosimeter irradiated with an MLC pencilbeam checkerboard pattern under lateral compression up to 27% to a non-deformed control dosimeter irradiated with the same pattern verified dose tracking under deformation. CTs of the experimental dosimeter prior to and during compression were exported into VelocityAI and used to map an Eclipse dose distribution calculated on the compressed dosimeter to its original shape. A comparison between the VelocityAI dose distribution and the distribution from the dosimeter showed field displacements up to 7.3 mm and up to a 175% difference in field dimensions. These results highlight the need for validating deformable dose mapping algorithms to ensure patient safety and quality of care.

## 1. Introduction

Use of deformable image registration algorithms has introduced a method of calculating cumulative dose feedback for organs of interest subject to internal organ motion [1, 2]. While many methods of deformable registration exist, there is currently no method of validating how well these algorithms meet the challenge of correctly mapping dose within a volume. This could be remedied, however, with a 3D dosimeter capable of capturing dose under deformation, thus providing a physical distribution for comparison [3–5]. In this study, we evaluate a novel deformable PRESAGE^®^ dosimeter and investigate the feasibility of applying this dosimeter to a new method for validating dose tracking algorithms in deforming tissues.

## 2. Methods

### 2.1. Materials and Dose Sensitivity

**Figure 1:**
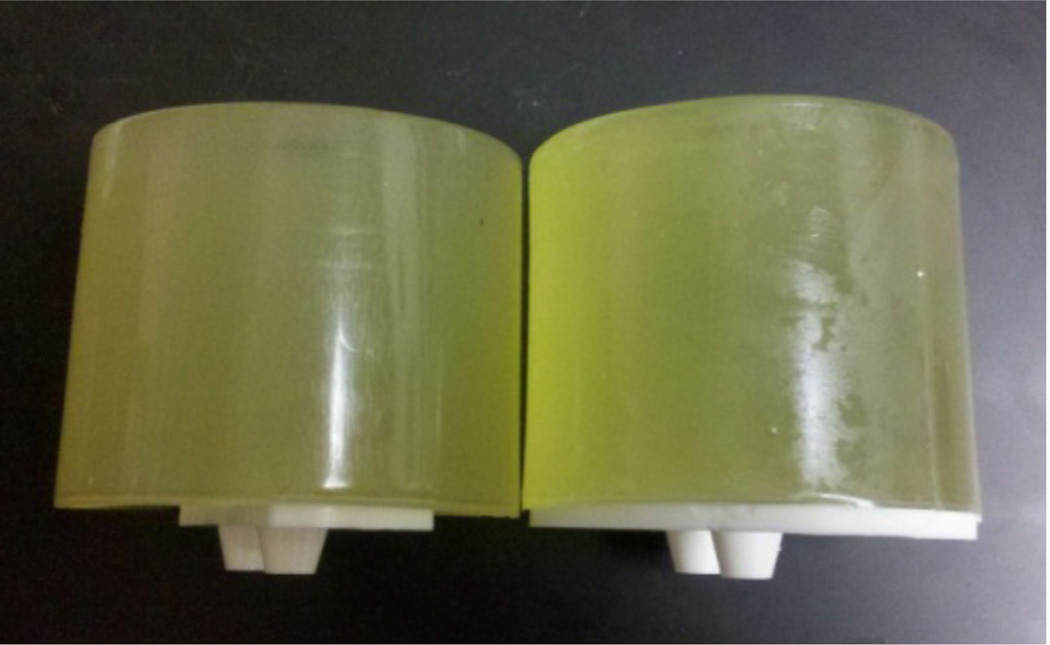
6 cm diameter cylindrical deformable dosimeters with attached plates keyed for directional registration in the optical-CT scanner.

Two cylindrical dosimeters (6 cm diameter, 4.75 cm long), as shown in Figure 1, were manufactured from a novel PRESAGE^®^ (Heuris Pharma, Skillman, NJ) formulation – consisting of an elastic polyurethane matrix doped with radiochromic leuco dye – which regains its original shape following physical deformation. This deformable PRESAGE^®^ formulation has a density of 1.02 g/cm, leuco dye content 2% (wt/wt) o-MeO-LMG, and Z_eff_ of 7.5. The Shore A hardness of the dosimeter is 10–20. Dose sensitivity was characterized by irradiating cuvettes containing PRESAGE^®^ material from the same batch to known doses up to 8 Gy.

### 2.2. Validating Quantitative Dose Tracking Under Deformation

Two 6 cm diameter cylindrical dosimeters were irradiated with a checkerboard arrangement of 5 mm square pencil beams created by MLC fields (Figure 3A). One dosimeter served as a control and was irradiated without deformation. The second, experimental dosimeter was irradiated under non-uniform lateral compression by up to 27% (1.6 cm) to simulate a deformed organ. Compression was removed after irradiation, allowing the dosimeter to return to its original shape.

High-resolution 3D dose distributions (isotropic 1 mm resolution) were obtained by optical-CT imaging [6]. Physical dose deformation was quantified by comparing pencil beam shapes and positions in the control and deformed dosimeters in MATLAB.

### 2.3. Application in Validating Deformation Dose Tracking Algorithm

**Figure 2:**
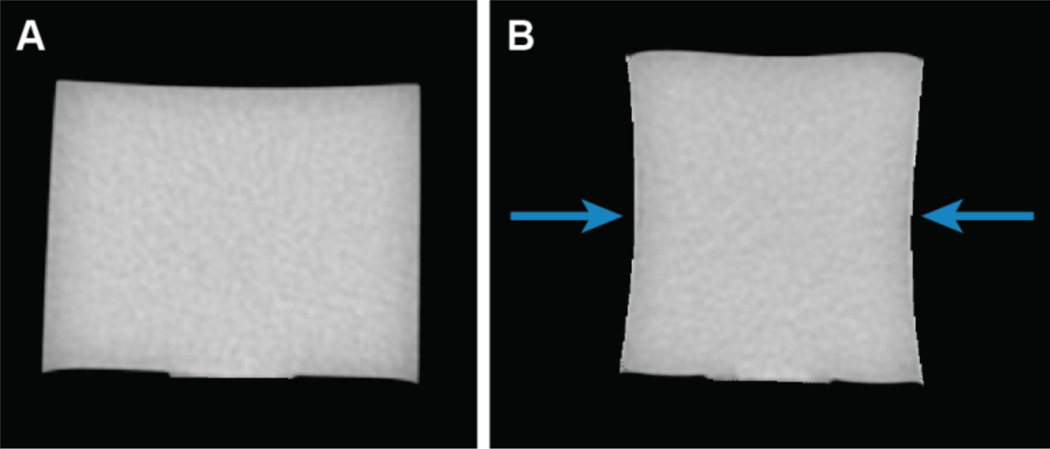
(A) CT of 6 cm diameter dosimeter without compression. (B) CT of the same dosimeter with compression up to 1.6 cm (27%). Arrows show direction of compression.

CT images taken of the experimental dosimeter both with and without compression (Figure 2) were imported into VelocityAI (Velocity Medical Solutions, Atlanta, GA), a deformation dose tracking software tool under evaluation in our clinic. The compressed dosimeter CT was deformed back to the shape of the uncompressed dosimeter using VelocityAI’s deformable image registration capabilities. This deformation was then applied to a dose distribution calculated on the CT of the compressed dosimeter (exported from Eclipse) to yield a deformed dose distribution for the dosimeter with compression removed.

The difference between the dose distribution from Velocity and our physical dosimeter data was determined at an axial slice in the region of greatest compression and quantified in MATLAB using 3 metrics: (1) the displacement of the centroid of each irradiated checker, and the differences in the full width at half maximum (FWHM) for a given checker along its (2) horizontal (along the axis of compression) and (3) vertical (perpendicular to the axis of compression) profiles.

## 3. Results

### 3.1. Deformable Formulation Characterization

The dose response of the deformable PRESAGE^®^ formulation across a range of 0 – 8 Gy demonstrated a linear response with a sensitivity of 0.0032 ΔOD/(Gy/cm).

### 3.2. Validation of Quantitative Dose Tracking Under Deformation

**Figure 3:**
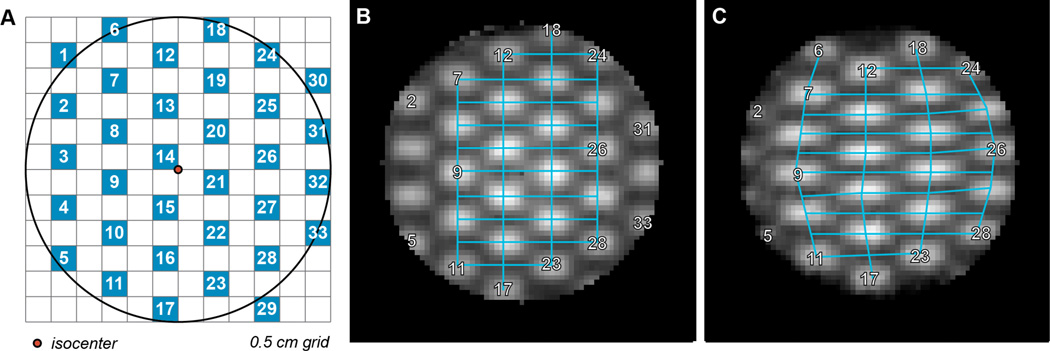
Geometric dose tracking in the deformed dosimeter is quantified with an overlaid grid. Edge artifacts from dosimeter optical-CT images (B-C) have been cropped. (A) Diagram illustrating the MLC checkerboard pattern. 5 mm×5 mm radiation fields are shown in blue. (B) Axial cross-section of an uncompressed dosimeter irradiated with the pattern in (A). (C) Axial cross-section of a dosimeter laterally compressed by 27% during irradiation with the same pattern.

A single slice of the optical-CT scan of the control (non-deformed dosimeter) is shown in Figure 3B. The corresponding slice through the second dosimeter, which was compressed laterally by 1.6 cm during irradiation, is shown in Figure 3C. This post-compression dose tracking is highlighted with overlaid gridlines that bisect the irradiated fields. Lateral compression by 27% resulted in expansion of the dose pattern by approximately 8% – 42% along the axis of compression and reduction of the dose pattern by approximately 11% – 13% perpendicular to compression. This clearly indicates that deformed dose tracking was successfully captured in the deformed dosimeter.

### 3.3. Comparison Between VelocityAI Deformation Algorithm and Deformable Dosimeter

**Figure 4:**
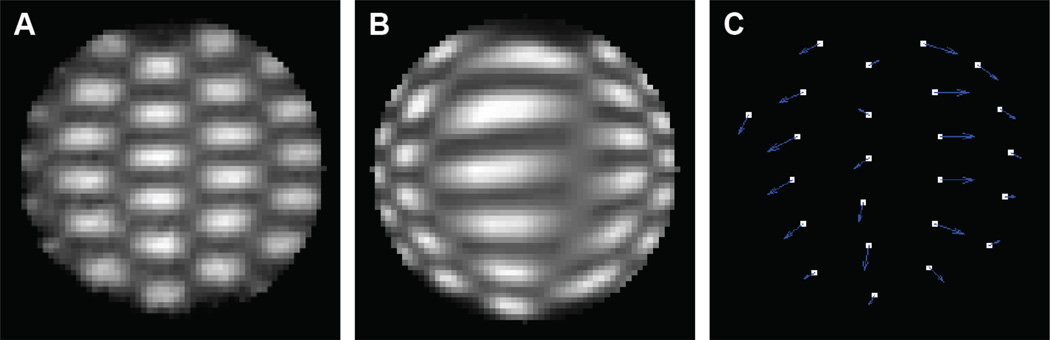
(A) Measured deformed dose distribution - axial cross-section of dosimeter laterally compressed by 27% during irradiation with the pattern shown in Figure 3A. (B) Corresponding axial cross-section showing the deformed dose distribution as predicted by VelocityAI deformable algorithm. (C) Quiver plot displaying magnitude of centroid displacement errors for each field in the VelocityAI distribution. White pixels mark the true location of centroids of each field in the dosimeter (i.e. positions in A).

Axial cross-sections of dose distributions in the region of greatest compression from the actual dosimeter and the VelocityAI deformation algorithm are shown in Figure 4A and 4B. Figure 4C displays the magnitude of displacement of each field’s centroid between the two. The displacement between VelocityAI’s calculated fields and the actual fields captured in the dosimeter ranged between 2 – 7.3 mm with a mean displacement of 4.5 mm.

The differences in field shapes between VelocityAI and the dosimeter were evaluated with a selection of representative fields and summarized in Table 1. Within this sample, horizontal FWHM (along the axis of compression) varied from 40% narrower to 175% wider than the actual fields. Vertical FWHM (perpendicular to the axis of compression) varied from 33.3% shorter to 50% taller than the actual fields. Magnitude of centroid displacement did not correlate with differences in field shape.

## 4. Conclusion

A novel, deformable 3D dosimeter was able to successfully track dose under deformation. These results are significant in that this deformable 3D dosimeter has potential to validate deformable dose tracking and registration algorithms. Applying this method to the deformable dose tracking algorithm in VelocityAI demonstrated significant limitations in accurately predicting deformable dose distributions within a homogenous structure. These results highlight the need for validating deformable dose mapping algorithms to ensure patient safety and quality of care.

## Figures and Tables

**Table 1:** Differences between the dose distributions from the Velocity AI deformable dose tracking algorithm and the physical dose tracking data from the deformable dosimeter.

Field^a^	Centroid Displacement^b^ (mm)	Horizontal FWHM		Vertical FWHM	

		Difference c (mm)	Percent Error^d^	Difference c (mm)	Percent Error^d^
9	5.8	−4	−40.0%	0	0.0%
13	2.2	14	175.0%	2	50.0%
17	2.2	1	12.5%	−1	−20.0%
18	7.3	10	125.0%	−2	−33.3%
21	7.0	2	22.2%	1	33.3%

a Field numbers refer to enumerated irradiated MLC fields as labelled in Figure 3D.

b Magnitude of displacement between the centroid of a given field in the Velocity distribution versus the corresponding field in the dosimeter.

c FWHM_Velocity_ – FWHM_dosimeter_

d (FWHM_Velocity_ – FWHM_dosimeter_)/FWHM_dosimeter_
